# Can trace element supplementations (Cu, Se, and Zn) enhance human immunity against COVID-19 and its new variants?

**DOI:** 10.1186/s43088-021-00123-w

**Published:** 2021-05-17

**Authors:** Bouzid Nedjimi

**Affiliations:** Laboratory of EVSE, Faculty of Science of Nature and Life, Ziane Achour University of Djelfa, P.O. Box 3117, Cité Aîn Chih, 17000 Djelfa, Algeria

**Keywords:** COVID-19, Essential trace elements, Human health, Immune system, New strains, SARS-CoV-2

## Abstract

**Background:**

Coronavirus-19 (SARS-CoV-2) is constantly changed through mutation, and new stains of this virus are detected throughout the world such as B.1.1.7 (UK), B.1.351 (South Africa), and P.1 (Brazil). These strains seem to be more easily transmissible than other variants, which may lead to more cases and more deaths. Currently, there are many vaccines for SARS-CoV-2 available in the market but without full clinical data beside. Despite the existence of these vaccines, the numbers of outpatients are still increasing in many countries around the world, and the reliability of these vaccines still remains elusive. It is well known that trace element deficiencies increase the individual susceptibility to immune dysfunction and lead to global health problem. In this context, improving the immune defense system to combats this pandemic is absolutely necessary. The purpose of this review is to establish the probable relation between trace elements supplementation and COVID-19.

**Main body:**

Several clinical studies confirmed that Cu, Se, and Zn insufficiencies alter the immune system and increase the vulnerability to viral infections. Based on antiviral and anti-inflammatory effects of these micronutrients, it seems logical that dietary supplementations of these components might enhance human immune system and lower the graveness of COVID-19 infection.

**Conclusion:**

Based on available data, we hypothesize that the clinical use of some essential trace element supplementations such as copper, selenium, and zinc might be a preventive and promising option to enhance human immunity against the new pandemic COVID-19 and its new strains.

**Graphical abstract:**

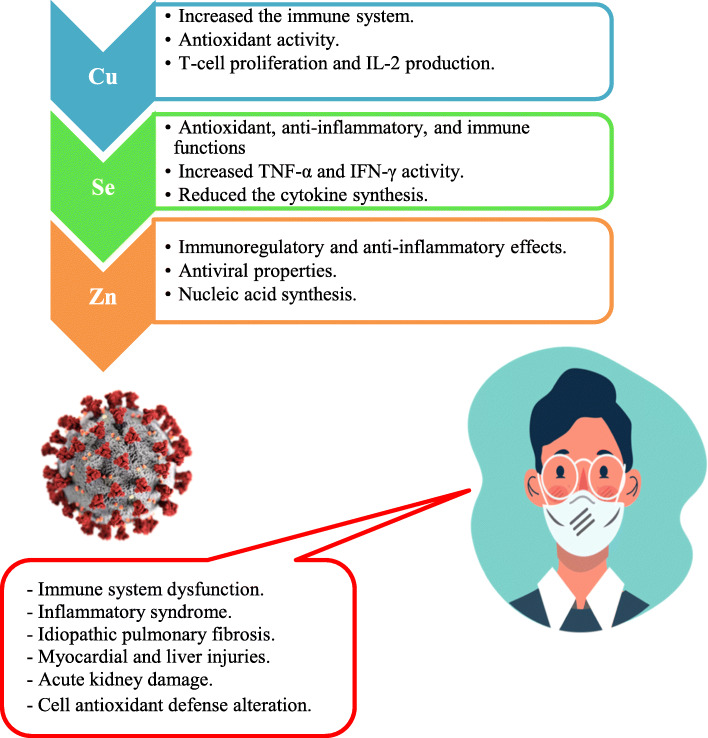

## Background

The common name “coronavirus” was used the first time in 1968 to classify a new family of viruses called *Coronaviridae* associated to single-stranded RNA genomes that cause respiratory diseases including influenza (common cold), severe acute respiratory syndrome (SARS-CoV), and middle east respiratory syndrome (MERS-CoV) [10]. There are 04 subfamilies of *Coronaviridae* including the alpha (α), beta (β), gamma (γ), and delta (δ) coronaviruses. These kinds of viruses signifying “crown” or “wreath”, which belong to the family of *Coronaviridae*, are spherical form enclosed in a triple envelopes (lipid and protein envelopes, and nucleocapsid) [20]. The diameter of the virus ranged between 80 and 120 nm with molecular weight of 40,000 kDa [18]. The external surface was covered by shaped spikes, from which its name comes [8].

According to the International Committee on Taxonomy of Viruses (ICTV), more than 40 kinds of coronaviruses were identified, with the vast majority detected in mammals. Seven coronaviruses can infect humans and among them three types of coronaviruses belong to the β-coronavirus subfamily and cause a high mortality rate: SARS-CoV, MERS-CoV, and SARS-CoV-2 (COVID-19) [10].

Coronavirus-19 (severe acute respiratory syndrome coronavirus) is a new grave pneumonic syndrome extended rapidly all over the world. This disease has a severe impact especially in elderly patients with hypertension, diabetes, and pneumatic diseases [36]. There are many vaccines trials for COVID-19 available in the market, with some positive but a reliable vaccine is not available yet. The SARS-CoV-2 is continually changing into novel emerging variants decreasing the efficiency of recent vaccines against virus in many countries in the world [12].

**Fig. 1:**
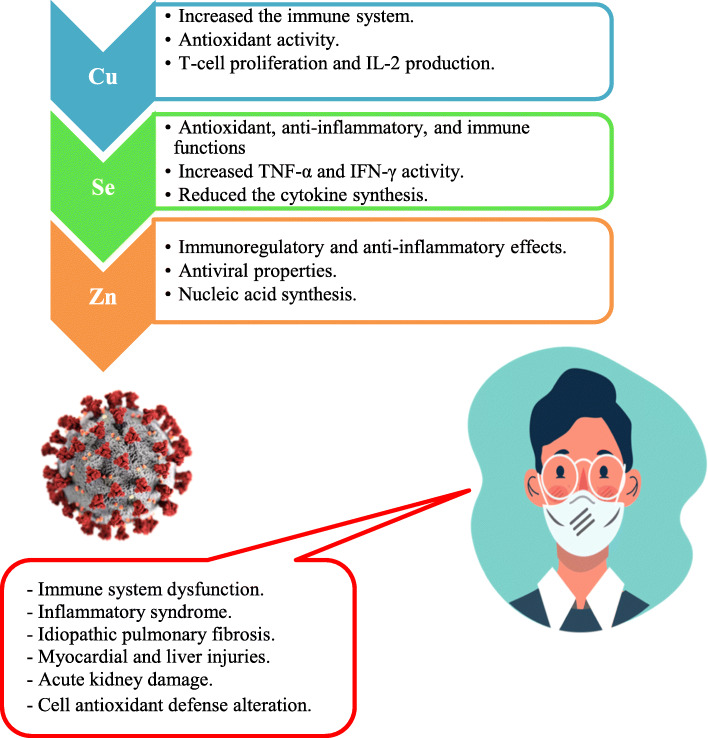
COVID-19 disorders and potential benefits of Cu, Se, and Zn supplementations

Currently, we have enough clinical information about the effect of pharmaceutical supplementation of trace elements concerning COVID-19 [9, 19, 27]. Dietary trace element supplementations might enhance human immune system and lower the graveness of the viral infection (Fig. 1). It should be mentioned that these low-cost and readily obtainable trace elements to treat viral infections have been largely ignored, due to scarce knowledge about their clinical aspects.

## Main text

Copper (Cu) plays a significant role in the immune system maintenance. This trace element is closely related to the blood immune cell functions such as natural killer (Nk) and T helper (Th) cells required in the viral infection elimination and antibodies synthesis. The recommended daily ingestion (RDI) of Cu is 0.90 mg/day [22] (Table 1). Cu acts as a cofactor of superoxide dismutase (SOD) involved in the cell defense from oxidative injury [16]. The antiviral activities of Cu in lytic virus replication by damaging RNA genome was approved by Ishida [14]. In the case of Cu shortage, the body shows an exceptional vulnerability to viral infections. Thus, Percival [23] demonstrated that immune dysfunction appear most pronounced in young and elderly patients with dietary Cu deficiency. Many viruses such as bronchitis virus, poliovirus, and virus type 1(HIV-1) were inactivated by Cu supplementation [26]. Hopkins and Failla [13] reported that Cu lack in human diet decreases the interleukin-2 (IL-2) synthesis and IL-2 mRNA of T-lymphocytes. According to Noyce et al. [21] the active viral particles of human influenza A virus decreased in number from 500,000 to 500 after incubation on Cu surface for 6 h. Warnes et al. [32] demonstrated that coronavirus 229E virus was morphologically affected (envelope degeneration, surface spikes disintegration, and genomic DNA damage) and remains inactive by Cu contact surfaces. In this way, recently, van Doremalen et al. [30] found that novel SARS-CoV-2 (COVID-19) is more sensitive to the Cu surface contact compared to SARS-CoV-1.

**Table 1:** Dietary sources, recommended daily intake (RDI), daily permissible limit (DPL), immune functions, and supplementation effects of Cu, Se, and Zn

Trace elements	Dietary source^**a**^	Content^**a**^	RDI^**b**^	DPL^**c**^	Immune functions	Deficiency	Supplementation effects	References
**Cu**	Beef meat	12.5 mg/100 g	0.9 mg/day	10 mg/day	- Supported macrophage function and natural killer (Nk) cell activity	-Decreased interleukin-2 (IL-2) production and IL-2 mRNA in T-lymphocytes	- Inhibited NF-kB activation - Increased IL-2 synthesis	- Percival [23] - Hopkins and Failla [13]
	Oysters, shellfish	4.9 mg/100 g						
	Mushroom	1 mg/100 g						
	Nuts	2 mg/100 g						
**Se**	Tuna	100 μg/100 g	55 μg/day	300 μg/day	- Increased T and B lymphocyte function - Increased the antioxidant enzyme activity	- Decreased Nk cell activity - Reduced the GSH-Px activity - Increased the expression of the inflammatory cytokine, IL-6, and TNF-α	-Improved immune function - Induced up-regulation of the IL-2 - Stimulated T-cell proliferation - Increased the TNF-α and IFN-γ	- Guillin et al. [11] - Alexander et al. [1] - Zhang et al. [35]
	Sardines	90 μg/100 g						
	Shellfish	85 μg/100 g						
	Chicken	25 μg/100 g						
	Eggs	20 μg/100 g						
	Nuts	1700 μg/100 g						
	Cereals	19 μg/100 g						
**Zn**	Oysters	61 mg/100 g	11 mg/day	40 mg/day	- Antiviral function - RNA polymerase inhibition - Enhanced the Nk cells activity - Activation of antibody production	- Induced viral infection vulnerability - Risk factor for pneumonia - Increased inflammation	- Reduced common cold severity - Increased the T-cell production	- Barnett et al. [2] - Read et al. [25] - de Almeida Brasiel [4]
	Beef	11 mg/100 g						
	Chicken	2 mg/100 g						
	Wheat	17 mg/100 g						
	Beans, lentils	1 mg/100 g						

^a^US Department of Agriculture [29]; European Food Safety Authority [7]

^b^Otten et al. [22]

^c^WHO/FAO [33]

Selenium (Se) acts as cofactor of many enzymes such as glutathione peroxidase (GPX) involved in the cell protection from oxidative damage (ROS). Otten [22] suggested that 55 μg of Se/day is sufficient dietary intake for human nutrition (Table 1). Poor Se status leads to increases the vulnerability to influenza virus infection [3]. The antiviral proprieties of selenite (Se^+4^) are related to antioxidant capacity of this trace element. Generally, the protein disulfide isomerase (PDI) of virus was blocked by the presence of selenite and leads to avoid virus to penetrate in healthy human cells [6]. Ivory et al. [15] demonstrated that Se supplementation has beneficial effects on immunity to influenza in older adults and enhanced T lymphocyte multiplication. Recently, Moghaddam et al. [19] showed that non-survivor SARS-CoV-19 patients have a pronounced deficit in total serum Se and SELENOP concentrations compared to surviving patients.

Zinc (Zn) is an essential trace element required for many metabolism activities. This component plays a substantial role in antiviral immunity, decreases the risk of viral infection, and possesses anti-inflammatory proprieties [24, 25]. Maares and Haase [17] shown that Zn regulates the lymphocyte apoptosis by regulating the cytokine activity. For human nutrition, optimal daily intake concentration of Zn is 11 mg/day [22]. Zn deficiency may be frequent for COVID-19 elderly patients [5]. te Velthuis et al. [28] revealed that Zn supplementation prevents the arterivirus RNA polymerase activity and blocks the coronavirus proliferation in vitro cell culture. In the USA, Barnett et al. [2] found that nursing home elderly have a significant T lymphocyte proliferation when supplemented by 30 mg Zn/day (Table 1). Also, Wang and Song [31] showed that Zn administration as an adjunct reduced the incidence and mortality of patients with severe pneumonia. In a recent clinical study, significant improvement was shown in 04 confirmed SARS-CoV-19 outpatients (26–63 years old) when treated with 115–184 mg Zn/day for 10 to 14 days [9]. In another clinical report, three SARS-CoV-19 patients (38–74 years old) recovered when they received 220 mg of Zn for 5 days, combined with hydroxychloroquine (HCQ) [27]. Contrary in observational study, Yao et al. [34] examined the remedy effects of zinc sulfate supplementation in 196 SARS-CoV-19 patients and indicated that Zn had a minimal effect on the survival of hospitalized patients.

Many countries throughout the world started clinical use of many vaccines such as Sputnik V, Pfizer-BioNTech, and Moderna and drugs like chloroquine (CQ), HCQ, remdesivir, lopinavir, and ritonavir to treat COVID-19, but without full clinical data beside. Numerous clinical investigations confirmed that trace element insufficiencies alter the immune system and increase the vulnerability to viral infections. For better enhancement of immunity including blood immune cell proliferation (lymphocyte apoptosis, natural killer (Nk) cells, T helper (Th) cells) and cytokine synthesis, sufficient daily intakes of essential trace element were required. We hypothesize that Cu, Se, and Zn supplementation can enhance immunity and reduced the risk of COVID-19 infection. Such information is essential to identification of new clinical trials to battle this pandemic.

## Conclusion

The new acute pulmonary infection (COVID-19) is usually associated with the decrease of human immune defense system leading to severe pneumonic inflammation and death. In view of the characteristics revealed above and the evidence of antiviral and anti-inflammatory effects of trace elements, it seems logical to assume that Se, Zn, and Cu could represent suitable components to strengthen immunity against viral infections including COVID-19 and its new emerging variants.

## Data Availability

All data generated or analyzed during this study are included in this article.

## Abbreviations

COVID-19: Coronavirus disease-2019
CQ: Chloroquine
DPL: Daily permissible limit
GSH-Px: Glutathione peroxidase
HCQ: Hydroxychloroquine
IFN-γ: Interferon-γ
IL-2: Interleukin-2
IL-6: Interleukin-6
MERS-CoV: Middle east respiratory syndrome
Nk: Natural killer cells
PDI: Protein disulfide isomerase
RDI: Recommended daily ingestion
ROS: Reactive oxygen species
SARS-CoV-2: Severe acute respiratory syndrome coronavirus 2
SOD: Superoxide dismutase
Th: T helper cells
TNF-α: Tumor necrosis factor-α
